# Reconstructive Techniques Following Malignant Eyelid Tumour Excision—Our Experience

**DOI:** 10.3390/jcm13206120

**Published:** 2024-10-14

**Authors:** Krzysztof Gąsiorowski, Michał Gontarz, Jakub Bargiel, Tomasz Marecik, Paweł Szczurowski, Grażyna Wyszyńska-Pawelec

**Affiliations:** Department of Cranio-Maxillofacial Surgery, Medical College, Jagiellonian University, 30-688 Cracow, Poland; michal.gontarz@uj.edu.pl (M.G.); jakub.bargiel@uj.edu.pl (J.B.); tomasz.marecik@uj.edu.pl (T.M.); pawel.szczurowski@uj.edu.pl (P.S.); grazyna.wyszynska-pawelec@uj.edu.pl (G.W.-P.)

**Keywords:** periocular malignancy, eyelid reconstruction, oculoplastic surgery

## Abstract

**Background**: Malignant eyelid tumours present a considerable challenge in the field of ophthalmic oncology, necessitating a combination of precision oncological care and meticulous reconstruction to ensure the preservation of eyelid functionality and the maintenance of facial aesthetics. **Method**: This study presents a review of the outcomes of 167 patients who underwent eyelid reconstruction following the excision of primary non-melanocytic malignant tumours. The choice of reconstruction technique was dependent on a number of factors, including the stage of the tumour, its location, and the characteristics of the patient. The most commonly used techniques included regional flaps, local flaps, and skin grafts. The most frequently employed reconstruction techniques were forehead flaps (59 cases), simple excisions (38 cases), and Mustarde cheek flaps (16 cases). **Result**: The postoperative complications, including ectropion, epiphora, and flap necrosis, were recorded. However, no significant correlation was found between the risk of complications and either the location of the tumour or the reconstruction method employed. Despite the complexity of medial canthal and lower eyelid reconstruction, satisfactory aesthetic and functional outcomes were generally achieved. **Conclusions**: This study emphasises the importance of individualised surgical planning, highlighting the advantages and limitations of various techniques to optimise both the functional and aesthetic results.

## 1. Introduction

Malignant eyelid tumours represent a significant challenge in ophthalmic oncology, not only because of their potential for local invasion and metastasis, but also because of the functional and aesthetic considerations inherent in this delicate and complicated anatomical region [1]. The eyelids play a crucial role in protecting the ocular surface, maintaining vision, and contributing to facial symmetry and expression [2]. Therefore, the surgical excision of malignant eyelid tumours requires careful planning and execution to ensure complete tumour removal while preserving as much of the function and appearance of the eyelid as possible [3].

Reconstruction after eyelid tumour excision is a complex and multifaceted process that requires a thorough understanding of the anatomy of the eyelid, the behaviour of different malignant lesions, and the available reconstructive techniques. The choice of a reconstruction method is influenced by several factors, including the size and location of the defect, the patient’s age and health status, and the need to balance oncological safety with functional and aesthetic outcomes [4].

The primary objective of this study is to review and evaluate the current approaches to eyelid reconstruction following the excision of malignant tumours. By comparing a range of techniques—from simple primary closure to more complex local and regional flaps—this study aims to identify the advantages and limitations of each approach. We hypothesise that a detailed analysis of these techniques will provide insights that could guide clinicians in selecting the most appropriate reconstructive methods based on individual patient factors and tumour characteristics.

Furthermore, this study aims to contribute to the existing literature by providing detailed information on surgical techniques, patient outcomes, and potential complications associated with eyelid reconstruction. We believe that our findings will help improve surgical planning and enable clinicians to better optimise functional and aesthetic outcomes for patients undergoing this complex and delicate procedure.

## 2. Materials and Methods

This retrospective study included patients who underwent surgery for primary malignant eyelid tumours at the Department of Cranio-Maxillofacial Surgery at Jagiellonian University in Krakow between 2002 and 2020. Patients who had not underwent reconstruction were excluded from this study, i.e., those who underwent exenteration with the post-excisional cavity left for granulation were excluded. In addition, patients with local recurrence who underwent surgery at both the local clinic and other centres were excluded from this study.

The database included the following: age, sex, location of the primary tumour focus, stage of the tumour, type of anaesthesia used during surgery, type of reconstruction performed, and complications. In this study, eyelid tumours were classified according to the American Joint Committee on Cancer (AJCC) 7th edition TNM staging system.

A decision regarding the reconstruction method was reached following a multidisciplinary medical consultation, taking into account a number of factors, including the patient’s age, overall health status, a histopathological examination, and the advancement of the tumour. Local flaps and grafts were the most frequently employed surgical techniques for the treatment of the less advanced tumours, whereas regional flaps were always utilised for more advanced tumours. In the patients with significant comorbidities, a reconstruction method that permitted the procedure to be performed under local anaesthesia was selected. In several cases, an intraoperative decision was made to change the reconstruction method due to the need to extend the procedure after obtaining a positive result from the intraoperative histopathological examination.

The analysis of the qualitative variables was performed by calculating the number and percentage of occurrences of each value. A comparison of the values of the qualitative variables in the groups was carried out using the Fisher’s exact test, because the expected numbers in the tables were low. A significance level of 0.05 was used for the analysis. Therefore, all *p*-values below 0.05 were interpreted as indicating significant relationships. The analysis was performed with the R program, version 4.1.3.

## 3. Results

This study included 167 patients who underwent surgery for primary non-melanocytic malignant tumours of the eyelid skin at the Department of Maxillofacial Surgery at the University Hospital in Krakow between 2002 and 2020. Of these, 83 were male and 84 were female. The mean age was 68 years. Table 1 shows the detailed data from the patients’ group.

The largest group consisted of patients with a diagnosed primary malignant tumour in the medial canthus of the eye (78 patients) and the lower eyelid (56 patients). Less common locations were as follows: upper eyelid, lateral canthus, and patients with tumours in more than one location.

**Figure 1:**
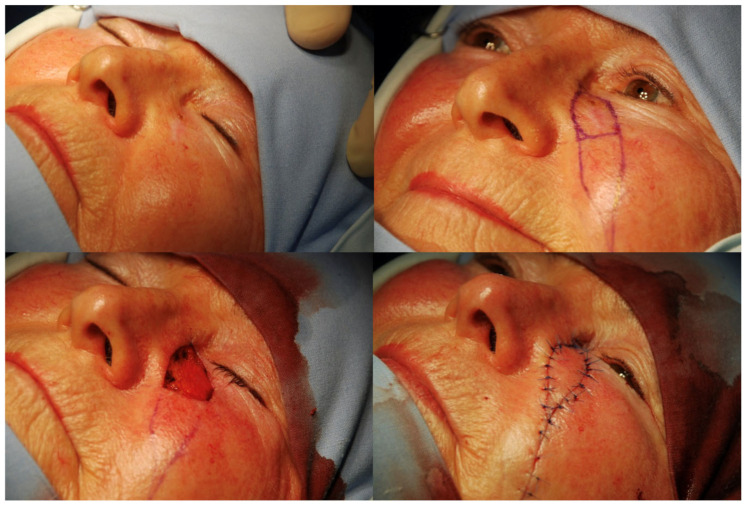
V-Y advancement flap.

The regional flaps were used most frequently for tumours in the medial canthus, and least frequently for tumours in the lateral canthus. Skin grafts were only used for tumours in the lower eyelid and very rarely for ones in the medial canthus. Local flaps and skin grafts were used most frequently for T1b and least frequently for T3b and T4a (Figure 1). Regional flaps were always used for T3b and T4a, and least commonly (not at all) used for T1b. In general, except for T1a, the higher the stage of progression, the more often a regional flap was used, and less often a local flap and skin graft were used (Table 2).

The most commonly used method of reconstruction was the forehead flap (59 patients), followed by a simple excision (38 patients), the glabellar flap (19 patients), and the Mustarde cheek flap (16 patients). Less commonly used reconstruction methods included the frontotemporal flap, full thickness skin graft, and the scalp flap, which was used in one patient with an advanced lesion.

In the less advanced cases, where the risk of distortion of the eyelid by suturing the excision was minimal, defects were managed by simple approximation. Specifically, this technique was applied to the defects involving less than a quarter of the total lid length in the younger patients and less than a third in the older patients. Care was taken to avoid excessive palpebral tension and to preserve the integrity and function of the eyelid. By following these guidelines, the optimal results were achieved and the likelihood of complications such as lid retraction or asymmetry was minimised.

There was no statistically significant correlation between tumour location and the risk of postoperative complications (*p* = 0.674) (Table 3).

There was also no statistically significant correlation between the method of reconstruction for the postoperative defect and the occurrence of complications. The higher the stage of advancement, the more often a regional flap was used and the less often a local flap or graft was used.

The most common complication after excision and reconstruction was lower lid ectropion, which occurred in 10 patients. The second most common complication was epiphora, which occurred in seven patients. Less common complications included partial necrosis of the flap or graft followed by deformation of the eyelid, requiring further contouring. The least common complication was wound suppuration.

One of the most common complications following the excision of malignant tumours of the lower eyelid was ectropion. Despite the frequency of ectropion in postoperative cases, no statistically significant correlation was found between the type of reconstructive technique used for lower eyelid reconstruction and the risk of ectropion development (*p* = 0.289) (Table 4).

**Figure 2:**
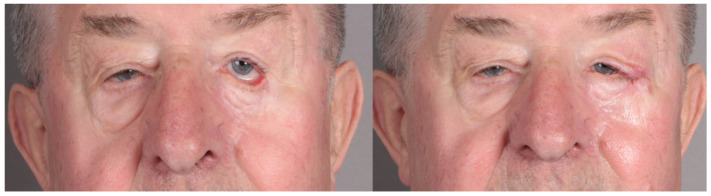
Surgical ectropion repair.

A surgical treatment for ectropion in our patients was typically performed within six to twelve months of the tumour resection. This time frame allowed for initial wound healing and a thorough assessment of the functional and aesthetic needs of the patient. The most common technique used to correct ectropion was the lateral tarsal strip procedure (Figure 2).

The second most common complication observed in this study was epiphora. This complication was only seen in patients undergoing surgery for advanced (>T2b) malignant tumours in the medial canthus where a frontal flap was used for reconstruction.

The reconstruction of the lacrimal drainage system included silicone stent intubation of the remaining canalicular system to maintain patency. However, in some cases, the reconstruction of the lacrimal drainage system was delayed until the final histopathological results confirmed a complete tumour excision with clear margins. This approach ensured that the reconstructive efforts would not be compromised by a residual malignancy.

In our study, recurrence occurred in six cases, with a median recurrence time of 26 months after the initial procedure. Management of the recurrences included an extension of the surgical resection in all cases. In addition, a regional flap was used for reconstruction in each case to ensure adequate coverage and to restore eyelid function. The aim of this approach was to achieve both oncological safety and satisfactory postoperative aesthetic and functional outcomes.

## 4. Discussion

Reconstructing the eyelid and periorbital area presents significant challenges due to the unique characteristics of the tissues in this region, which are notably thin, elastic, and mobile. In contrast, the tissues commonly used for reconstruction, typically harvested from areas closer to the orbit, tend to be thicker and less pliable [1].

According to the Mustarde rule, defects involving less than a quarter of the total lid length in younger patients and less than a third in older patients can usually be managed with simple approximation, taking care to avoid excessive palpebral tension; a lateral canthotomy may be performed if necessary [5]. However, for larger, full-thickness defects, extending from one-third to one-half of the lid length, a local flap is usually required to reconstruct the anterior lamella, with a cartilage or mucosa graft used for the posterior lamella [4,6,7].

Local flaps are often the initial surgical approach of choice for the repair of minor defects of the eyelids and surrounding eye region. Local flaps are widely used due to their ability to achieve aesthetically pleasing results [8,9]. This is largely due to the superior match they provide in terms of skin pigmentation, texture, and the natural structural properties of the eyelid tissue. In addition, the robust vascular supply to these flaps increases their reliability, resulting in a lower incidence of complications such as flap necrosis or delayed healing [10]. In addition, the use of local flaps eliminates the need for complex microvascular surgical techniques, making them a simpler and more effective option for many patients [11].

The choice of reconstruction method is influenced by factors such as the precise location and size of the excised lesion, the specific characteristics of the tumour, and the patient’s age [4,5]. When planning the appropriate method of eyelid defect reconstruction, it is important to consider not only the histopathological findings of the initial biopsy, but also the need for a wide excision of the lesion and the potential need for an extended resection if the intraoperative histopathological results indicate positive margins. This approach is supported by the findings of Gąsiorowski et al., who demonstrated that the risk of an incomplete excision and consequently local recurrence is almost five times higher in the aggressive subtypes of basal cell carcinoma (BCC). These data highlight the importance of carefully selecting reconstruction techniques that allow for the possibility of extended surgical margins to ensure oncological safety and minimise the risk of recurrence [12].

In some cases of malignant eyelid tumours, reconstruction may not always be feasible due to the extent of resection required to achieve clear margins. Advanced tumours, especially those invading the orbit or involving critical anatomical structures such as the lacrimal system or canthal tendons, often require more aggressive surgical approaches, including orbital exenteration with delayed reconstruction [13].

In periorbital reconstructive planning, the primary goal is to achieve functional and aesthetic outcomes. Specifically, at the medial canthus the goal is to preserve the natural concavity without distorting the surrounding tissues [14]. Maintaining a normal eyebrow contour and symmetry is essential as scar contracture can lead to unsightly webbing or scarring ectropion, causing secondary epiphora [10].

Reconstructive surgery for the medial canthus is considered to be one of the most challenging procedures due to its complex anatomy and aesthetic implications. The medial canthal region plays a pivotal role in determining the shape and appearance of the eye, and even minor deformities or asymmetries are readily apparent [2,4,15].

Defects in the medial canthal region are best treated with local or loco-regional flaps. For smaller defects in the upper part, the glabellar flap is the preferred choice due to its proximity and tissue characteristics [3,16]. For defects in the lower part, flaps harvested from the nasolabial area are generally more effective. For larger defects that cannot be corrected with a single flap, a combination of flaps may be used, such as the glabellar or forehead flap together with a cheek rotation flap. This approach is known to provide satisfactory aesthetic and functional results [2,17].

A variety of techniques have been described to reconstruct defects in this area. These include skin grafts, local/loco-regional flaps such as the rhomboid flap, glabellar flaps, bilobed flaps, myocutaneous V-Y advancement flaps, pickaxe double flaps, forehead flaps, and eyelid myocutaneous flaps [3,4].

In this study, the most commonly used methods for medial canthal reconstruction were the forehead flap, used in forty cases, and the glabellar flap, used in eight cases. The two-stage approach required for medial canthal reconstruction allowed for additional skin contouring in the medial canthal area.

**Figure 3:**
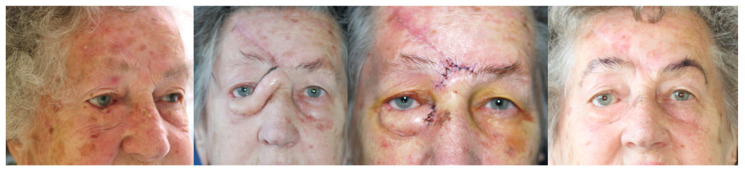
Forehead flap.

Forehead flaps are an excellent choice for the reconstruction of large and deep defects in the medial canthal region extending into the eyelid, particularly when defects are too large for eyelid flaps but not extensive enough to require free flaps (Figure 3). The paramedian forehead flap (PMFF) is particularly effective due to its robust vascularisation, mainly supplied by the supratrochlear artery, which allows a relatively narrow pedicle to support a large or long flap [18]. The proximity of the flap ensures a harmonious match in skin colour, tissue texture and structural congruence between the donor and recipient sites, making it ideal for periorbital reconstructions, including those associated with nasal defects [19]. It is often used in conjunction with other local and regional flaps or grafts to address significant periorbital defects. However, the technique requires careful planning to minimise complications such as distal flap necrosis, haematoma formation and suboptimal aesthetic results. These complications can be reduced by creating an appropriately sized flap (no less than 1.0–1.2 cm at the base), ensuring a tension-free closure, avoiding excessive thinning and maintaining meticulous haemostasis throughout the procedure. Despite its advantages, the forehead flap has some disadvantages, including the need for a two-stage procedure, potential colour mismatch, bulkiness and scarring at the donor site [20,21].

The glabellar flap technique is widely considered to be the most appropriate method for addressing significant defects involving the medial canthal area following tumour removal. The glabellar flap is often chosen due to its ability to mimic the colour, texture and thickness of adjacent tissue, resulting in unobtrusive scars [22]. However, a potential limitation of using the glabellar flap for medial canthal reconstruction is the risk of developing a bulky nasal dorsum due to the thick skin and subcutaneous tissue present in the glabella region [23]. Another disadvantage of the glabellar flap technique is that it has a tendency to bring the eyebrows closer together, as the bridge of the nose where the flap is placed becomes the stalk of the eyebrow [24].

However, Bertelmann et al. have described a modification of the glabellar flap for medial canthal reconstruction, suggesting a tunnelling technique to prevent the potential development of a bulge over the dorsum of the nose [15].

The reconstruction of the lower eyelid after a tumour excision presents significant challenges. The method chosen for reconstruction depends on several factors, such as the specific location and size of the lesion removed, the age of the patient, and the specific characteristics of the tumour [4,25]. Full-thickness defects involving a quarter to a third of the lid length are amenable to primary closure [3]. However, full-thickness defects extending between one-third and one-half of the eyelid length typically require a local flap to reconstruct the anterior lamella, accompanied by either a cartilage or mucosal graft for the posterior lamella [26].

The goal of lower eyelid repair is to preserve the essential functional components, including maintaining an intact tear–corneal interface, ensuring complete eyelid closure, preventing visual field obstructions, minimising scarring, and, when feasible, achieving aesthetic goals by concealing scars [1,3,4]. The approach to repairing a lower eyelid defect typically follows the reconstructive ladder and includes primary closure, grafts, and/or local flaps.

**Figure 4:**
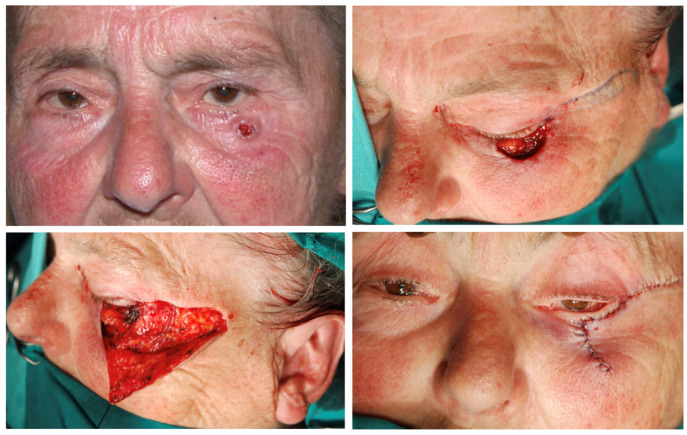
Cheek rotation skin (Mustarde) flap.

In this study, the most commonly used methods for lower eyelid reconstruction included the median forehead flap, the Mustarde cheek flap, and a simple excision with cantholysis (Figure 4).

In cases requiring full-thickness eyelid reconstruction, auricular cartilage grafts were used to restore the tarsal framework, while the inner eyelid layer was reconstructed using an oral mucosa graft. This approach is considered the standard for eyelid reconstruction and has been used successfully by other authors such as Pushker et al. and Yamamoto, demonstrating its reliability and efficacy in restoring both functional and aesthetic results [6,7].

The reconstruction of the upper eyelid following the excision of malignant skin tumours is a highly complex procedure that is critical for both functional and aesthetic outcomes. The upper eyelid plays a vital role in protecting the cornea as it covers most of its surface area, shielding the eye from dryness and potential damage. Therefore, its reconstruction is of paramount importance. Achieving a successful result requires the creation of a mobile eyelid that provides optimal corneal protection while maintaining an aesthetically pleasing appearance. Full-thickness defects greater than 25% of the upper eyelid width cannot be closed directly and require the use of local flaps [27]. Techniques such as superficial temporal artery-based flaps or those harvested from the forehead region are commonly used, providing the necessary skin and subcutaneous tissue to restore the missing eyelid segment [28].

In this study, the most commonly used method for upper eyelid reconstruction was the Fricke flap. For superficial post-resection defects, simpler techniques such as a direct closure with cantholysis or skin grafting were used. These methods allowed for the effective restoration of eyelid function and appearance. The Fricke flap provided robust coverage in cases of extensive tissue loss, whereas a direct closure and skin grafts were suitable for smaller, less complex defects [29].

In this study, no cases of ptosis were observed following the excision of upper eyelid malignancies. However, ptosis remains a recognised complication of eyelid reconstruction due to the impaired function of the levator palpebrae superioris muscle. Several surgical techniques have been described to manage ptosis, especially its congenital form, including a frontalis sling, levator advancement, Whitnall sling, frontalis muscle flap, and mullerectomy [30]. The choice of technique depends on the severity of the patient’s ptosis and the degree of levator function.

The reconstruction of the lateral canthus is a complex and important aspect of eyelid surgery, as this structure plays a key role in both the functional and aesthetic integrity of the eye.

In the reconstruction of lateral canthal defects following the excision of malignant eyelid tumours, the most commonly used technique in this study was S-plasty. This method allows for the effective realignment of tissue while minimising the tension and scar visibility. A critical aspect of this procedure was ensuring the proper repositioning of the lateral canthal tendon, as it plays a key role in maintaining the functional and aesthetic integrity of the eyelid [1].

In addition, it significantly influences the shape and appearance of the palpebral fissure, contributing to the ethnic characteristics of an individual [2]. Damage to this delicate area can lead to serious complications, including poor aesthetic outcomes, incomplete lid closure, inadequate corneal protection, and excessive tearing.

When local skin flaps are not viable for eyelid reconstruction, the use of free flaps may be an option. There are limited reports of employing microvascular free flaps in patients with complete eyelid loss but an intact globe. Rubino et al. described the application of a perforator anterolateral thigh flap, with anastomosis to the superficial temporal vessels and the inclusion of a fascial strip for structural support [31]. Thai et al. reported the use of a dorsalis pedis flap combined with nasal septal cartilage in a burn patient [32].

Surgical excision remains the most common treatment for patients with non-melanoma skin cancer (NMSC). However, individuals with advanced-stage NMSC are often not suitable candidates for surgery and, if surgery is performed, they have a higher risk of a post-surgical recurrence. Recent research has focused on addressing this issue by developing targeted therapies and immunotherapies. The dysregulated intracellular signalling pathways in NMSC have been identified as potential therapeutic targets. Specifically, the hedgehog signalling pathway has been targeted for basal cell carcinoma (BCC), while the epidermal growth factor receptor (EGFR) is a key target in cutaneous squamous cell carcinoma (cSCC). Additionally, immunotherapy has shown promise in treating systemic disease through the immune checkpoint inhibitors targeting the PD-1/PD-L1 axis and CTLA-4 [33]

Radiotherapy represents an efficacious and adaptable non-surgical modality for tissue conservation, offering a valuable alternative for patients where excision may be either infeasible or result in suboptimal aesthetic outcomes. Furthermore, adjuvant radiotherapy following surgery can assist in reducing the risk of recurrence and the associated morbidity, particularly in patients with unfavourable histological findings [34]. In a phase II study conducted to assess the response of older patients with early-stage non-melanoma skin cancer (NMSC) to accelerated radiotherapy, 30 out of 31 participants demonstrated complete responses. The study concluded that over 90% of patients achieved local disease control, which is considered a favourable outcome [35].

## 5. Conclusions

When planning the reconstruction of eyelid defects following the excision of malignant tumours, it is important to consider the specific location of the defect. For lower eyelid defects, the primary concern is maintaining proper lid margin alignment to prevent ectropion and ensure adequate ocular surface protection. Upper eyelid defects require careful attention to the levator mechanism and the preservation of lid function, particularly to avoid ptosis and to ensure a sufficient blink reflex for corneal protection. Medial canthal defects may involve the nasolacrimal system, requiring techniques to maintain tear drainage and avoid complications such as epiphora. Lateral canthal defects require the preservation of the lateral canthal tendon to maintain lid position and symmetry. Finally, the extent of the defect—whether partial or full-thickness—determines the complexity of the reconstruction, ranging from a direct closure for small defects to local flaps, grafts, or even staged procedures for larger or more complex defects to restore both function and aesthetics. Further research should focus on refining these approaches and identifying the optimal strategies for specific defect types and patient populations.

In summary, there are a number of techniques available for periocular reconstruction. With so many options to achieve the same goal, it is safe to assume that none of them is optimal. However, it is important to be aware of the different techniques that can be used for eyelid reconstruction, as different procedures may be required depending on the location and size of the defect. Multiple surgeries may be required to achieve the desired results, addressing first the loss of tissue and then the loss of function.

## Figures and Tables

**Table 1:** Overview of the types of reconstructions, tumour locations and complications.

Types of Reconstruction Methods Used	No of Patients
Forehead flap	59
Simple excision with cantholysis	38
Skin graft	11
Mustarde cheek flap	16
Frontotemporal flap	10
Cutler-Beard bridge flap	1
S-plasty	7
V-Y advancement flap	5
Glabellar flap	19
Converse flap	1
Tumour location	
Medial canthus	78
Lower eyelid	56
Upper eyelid	11
Lateral canthus	8
Medial canthus and lower eyelid	10
Medial canthus and both eyelids	2
Medial canthus and upper eyelid	1
Lateral canthus and upper eyelid	1
Complications Observed	
Tearing	4
Suppuration	1
Extropion	7
Deformation	2
Flap necrosis	4
Tearing and ectropion	2
Ectropion and deformation	1
Tearing and flap necrosis	1

**Table 2:** The correlation between the tumour stage and method of reconstruction used.

Method of Reconstruction	Primary Tumour (T)						*p*
	1a (*n* = 7)	1b (*n* = 6)	2a (*n* = 33)	2b (*n* = 48)	3a (*n* = 32)	3b, 4a (*n* = 10)	
Local flap	4 (57.14%)	5 (83.33%)	16 (48.48%)	17 (35.42%)	3 (9.38%)	0 (0.00%)	*p* < 0.001
Regional flap	3 (42.86%)	0 (0.00%)	14 (42.42%)	28 (58.33%)	28 (87.50%)	10 (100.00%)	
Skin graft	0 (0.00%)	1 (16.67%)	3 (9.09%)	3 (6.25%)	1 (3.12%)	0 (0.00%)	

**Table 3:** The correlation between tumour location and the risk of local complications.

Complications	Localization of Tumour						*p*
	Medial Canthus (*n* = 78)	Lower Eyelid (*n* = 56)	Upper Eyelid (*n* = 11)	Lateral Canthus (*n* = 8)	Medial Canthus and Lower Eyelid (*n* = 10)	Other (*n* = 4)	
No	69 (88.46%)	46 (82.14%)	10 (90.91%)	8 (100.00%)	9 (90.00%)	3 (75.00%)	*p* = 0.674
Yes	9 (11.54%)	10 (17.86%)	1 (9.09%)	0 (0.00%)	1 (10.00%)	1 (25.00%)	

**Table 4:** The correlation between the reconstruction method and the occurrence of complications.

Complications	Methods of Reconstruction			*p*
	Local Flap (*n* = 55)	Regional Flap (*n* = 102)	Skin Graft (*n* = 8)	
No	50 (91.11%)	82 (80.72%)	7 (87.50%)	*p* = 0.289
Yes	5 (8.89%)	20 (19.28%)	1 (12.50%)	

## Data Availability

Restrictions apply to the availability of these data. Data were obtained from patients treated at the Department of Cranio-Maxillofacial Surgery, Cracow, Poland, and cannot be shared, in accordance with the General Data Protection Regulation (EU) 2016/679.
